# The Paradox of Calcium Silicate‐Based Sealers: A Reflection on Bioactivity, Solubility and Clinical Meaning

**DOI:** 10.1111/iej.70083

**Published:** 2025-12-17

**Authors:** Emmanuel João Nogueira Leal da Silva, Marco A. Versiani

**Affiliations:** ^1^ Department of Endodontics Rio de Janeiro State University Rio de Janeiro Brazil; ^2^ Department of Endodontics Fluminense Federal University Niterói Brazil; ^3^ Grande Rio University (UNIGRANRIO) Rio de Janeiro Brazil; ^4^ Dental Specialty Centre Brazilian Military Police Belo Horizonte Minas Gerais Brazil

**Keywords:** bioactivity, calcium silicate‐based sealers, endodontics, root canal sealer, solubility

## 1 Introduction

When calcium silicate‐based root canal sealers (CSS) were first brought into clinical use in 2008 (U.S. Food and Drug Administration 2023), they were viewed as the beginning of a shift toward more biologically guided filling materials. Derived from mineral trioxide aggregate but refined for improved handling, these materials seemed to provide clinicians with the solution they had been looking for: an injectable, ready‐to‐use sealer capable of setting and hardening in moist conditions, simplifying filling while maintaining biological function. Overtime, this uncommon combination of advantageous physicochemical characteristics and remarkable biological potential contributed significantly to their growing acceptance among clinicians worldwide (Guivarc'h et al. 2020). However, it is worth remembering that moving a new endodontic sealer from a promising innovation to a clear understanding of its long‐term performance is rarely straightforward (Barborka et al. 2017). Accordingly, nearly two decades after their introduction, the early impression of balance and reliability associated with CSS now seems much less clear.

Today, the endodontic community faces many unanswered questions, as the expected benefits of these materials are increasingly challenged by clinical observations showing a worrying pattern. Preliminary clinical impressions presented in scientific meetings or informally discussed among clinicians often include follow‐up exams where CSS appear significantly reduced or even nearly absent from the root canal, especially in the apical third. These findings suggest that this is not a rare occurrence, but a pattern seen across different practitioners and clinical situations. What were once isolated observations are now converging into a consistent trend, prompting a more careful examination of how these materials behave inside the tooth and questioning previous assumptions about their long‐term stability. These clinical impressions, although not yet fully supported by long‐term clinical trials, prompt an important question: are CSS behaving differently inside the tooth than originally expected based on laboratory studies? If this is the case, it becomes necessary to reconsider how sealer performance should be interpreted and which criteria are most appropriate for assessing their behaviour over time. Therefore, rather than attempting to solve these questions directly, this paper examines how solubility, bioactivity, and long‐term stability coexist and interact in CSS and reflects on how these interactions challenge established concepts, commonly used testing methods, and current clinical expectations.

## 2 Limitations of Current Standards

In endodontics, it has been widely recognised that the primary function of filling materials is to create a long‐lasting seal of the root canal system, thereby isolating any remaining microorganisms and preventing their recolonisation (Tait et al. 2025). Among the physicochemical properties that determine sealer performance, dimensional stability and solubility are particularly critical because they directly affect the ability of the material to maintain structural integrity and preserve the sealing of the root canal system over time (De‐Deus et al. 2022). Dimensional stability refers to the capacity of a sealer to maintain its original form after setting and subsequent immersion in water, while solubility describes the extent to which the material dissolves when in contact with fluids over time (American Dental Association 2021; International Organization for Standardization 2025).

Several publications reported that some CSS meet international requirements for flow, setting time, radiopacity, and dimensional stability, and show minimal changes in volume after immersion in water or simulated body fluids (Ferreira et al. 2022; Zamparini et al. 2022; Kwak et al. 2023; Quaresma et al. 2024; de Sales Oliveira Neto et al. 2025). However, other studies have shown that certain CSS formulations may not set properly under controlled laboratory conditions (Loushine et al. 2011; Xuereb et al. 2015; Prullage et al. 2016; Lee et al. 2017; Silva et al. 2021), a concern further reinforced by reports of significant material loss, sometimes exceeding 30% over a short testing period, regardless of whether the tests are performed in distilled water or phosphate buffered saline (Siboni et al. 2017; Elyassi et al. 2019; Zordan‐Bronzel et al. 2019, 2021; Torres et al. 2020; Donnermeyer et al. 2022; Kim et al. 2024; Quaresma et al. 2024; de Sales Oliveira Neto et al. 2025; Scardini et al. 2025). Only a few experimental formulations with specific proportions of calcium silicate and radiopacifiers have shown solubility values within the acceptable ISO range (de Sales Oliveira Neto et al. 2025), suggesting that solubility may display formulation‐dependent patterns in the context of CSS, and raising real concerns about the long‐term stability of these materials. On the other hand, given the inherent behaviour of hydraulic materials like CSS, it has been argued that this issue may arise not only from the materials themselves but also from methodological limitations (Gandolfi et al. 2015; Siboni et al. 2017; Elyassi et al. 2019).

The international standard tests for evaluating the solubility and dimensional stability of root canal sealers usually involve placing set samples in water. While this method helps ensure consistency, it only provides a simplified view of real clinical conditions and does not capture the biological complexity of the in vivo environment (Aminoshariae et al. 2022; De‐Deus et al. 2022). In fact, the current standards provide a pass/fail threshold but do not offer a quantitative framework to distinguish between an ‘acceptable’ level of dissolution that supports bioactivity and a ‘harmful’ level that compromises the long‐term seal. This raises an important concern, as standard tests designed to ensure uniformity and reproducibility may also limit our understanding of materials that naturally behave in more complex biological contexts. In reality, hydraulic materials are regarded as active and interact continuously with their environment. When placed in the root canal, the calcium hydroxide released by the CSS interacts with dentinal substrates, tissue fluids, and blood, promoting the precipitation of calcium phosphate and calcium carbonate phases on the material surface. These interfacial reactions modify the surface chemistry and microstructure of the sealer, thereby influencing its apparent solubility and potentially playing a critical role in its biological behaviour and bioactivity (Camilleri 2007).

## 3 The Paradox—Bioactivity Versus Stability

CSS release ions through hydration of their calcium silicate phases, which liberates calcium and hydroxide ions that raise the local pH and drive bioactivity (Lopez‐Garcia et al. 2020; Sanz et al. 2021; Zamparini et al. 2022; Quaresma et al. 2024; Liu et al. 2025; Radwanski et al. 2025). Hydration of tricalcium and dicalcium silicate forms calcium hydroxide and a calcium silicate hydrate gel, and the dissociation of calcium hydroxide produces sustained alkaline pH values often above 11 for several weeks (Kwak et al. 2023; Liu et al. 2025). Ion release depends on phase composition, porosity, and additives, with higher open pore volume and specific formulations promoting greater calcium diffusion (Zamparini et al. 2022; Dimitrova et al. 2024). The released calcium supports calcium phosphate nucleation and may yield a mineralized surface layer under physiological conditions, although the degree of hydroxyapatite formation varies among products and can be reduced by carbonation (Sanz et al. 2021; Zamparini et al. 2022; Radwanski et al. 2025). These processes increase local pH, enhance antibacterial effects, and stimulate osteogenic and cementogenic gene expression in periodontal ligament cells, thereby supporting healing and regeneration (Lopez‐Garcia et al. 2020; Sanz et al. 2021). This biological response aligns with the strong positive correlation between calcium release and pH that has been consistently reported across different commercial sealers (Liu et al. 2025).

The complexity of CSS behaviour helps explain a paradox in the scientific literature, where the same community that questions the validity of solubility tests often accepts similarly artificial in vitro models when assessing cytotoxicity or bioactivity. In those settings, cell cultures exposed to material extracts are widely regarded as reliable indicators of biological behaviour. Under these conditions, CSS consistently shows low cytotoxicity and remarkable osteoinductive potential, while epoxy resin‐based sealers, for example, generally perform less favourably in the same models. The irony becomes evident when a method considered inadequate in one context is accepted in another, depending on whether its outcomes support or challenge prevailing expectations. This duality highlights not only the complexity of these materials but also the subtle biases that influence our scientific judgement. It reveals the fragile boundary between empirical observation and interpretative belief, reminding us that data, regardless of how rigorously it is obtained, does not speak on its own. Its meaning is ultimately assigned by us, the scientists and clinicians who interpret it. Viewed from this perspective, CSS plays a role that goes beyond serving as filling materials. They reveal both the progress made in endodontic science and the contradictions that still remain. How these materials behave, whether as expected or in unexpected ways, forces us to reconsider established ideas, question our testing methods, and acknowledge that our own interpretations influence how we understand the development of the field.

Perhaps one of the most important paradoxes involving CSS relates to the very property that has prompted both enthusiasm and caution: their solubility. It is possible that the same characteristic often criticised for weakening dimensional stability may also support their biological activity. The dissolution and ion release that occur over time may not represent a flaw but rather a functional mechanism that enables these materials to interact with surrounding tissues. If their solubility were reduced, their beneficial effects might remain restricted, limiting the broader biological responses that set them apart from more inert materials. From this viewpoint, solubility may be understood not simply as a weakness, but as an essential part of how these materials function in a biological environment. This idea, however, leads to important reflections: What should we ultimately expect from an endodontic sealer? Should dimensional stability be the primary goal, or should biological interaction be valued to the same extent? Do we prefer a material that stays structurally unchanged but offers little biological activity, or one that stimulates beneficial responses such as osteoinduction, even if it gradually dissolves over time? These questions challenge traditional assumptions about what an ‘ideal’ filling material should be and prompt us to reconsider whether durability alone should represent progress.

This reflection becomes even more relevant when apical periodontitis is present. In this condition, it is often claimed that CSS can speed up the healing process by stimulating the body's response. However, when examined through rigorous clinical studies, the perception of faster repair with CSS becomes less convincing, as reported success rates are comparable to those achieved with other types of sealers (Sabeti et al. 2024; Zamparini et al. 2024; Kangseng et al. 2025). This observation aligns with clinical and biological evidence showing that when proper endodontic treatment is performed and the bacterial load is reduced to levels below the threshold that can interfere with tissue repair, healing is likely to occur naturally through the body's own regenerative processes, without the need for additional biological stimulation. This suggests that the idea of faster healing may be influenced more by expectations than by solid scientific evidence, creating real doubt about whether added bioactivity is truly needed or if it only speeds up a process that would happen naturally.

This line of thought introduces another paradox, suggesting that if CSS appear to promote faster healing, this effect may stem from their tendency to dissolve, which increases the release of bioactive components into the surrounding tissues. Earlier laboratory studies support this view, showing that their high solubility, a property usually considered to enhance the material's biological activity, may in fact be the underlying cause of that effect. In other words, the ability of CSS to dissolve and release ions is not just a side effect, but a key mechanism through which they interact with surrounding tissues and promote biological responses. However, while gradual material dissolution may support tissue repair, it can also weaken the long‐term integrity of the seal, increasing the risk of failure (Donnermeyer et al. 2019). This process of sealer washout may gradually expose the root canal interface, allowing microorganisms to become active again and potentially leading to periapical inflammation. It is important to emphasise, however, that this remains a hypothesis, since available clinical studies have not yet provided conclusive evidence that this mechanism can compromise the long‐term outcome of CSS.

Although this hypothesis has not yet been directly confirmed, it raises a biologically plausible concern supported by a previous report (Vieira et al. 2012). In this report, a tooth with apical periodontitis exhibited complete radiographic healing after retreatment but developed recurrent disease more than a decade later. The authors documented progressive dissolution and resorption of the zinc oxide‐based sealer, accompanied by fluid percolation and loss of sealing ability, which allowed dormant intratubular bacteria to regain access to nutrients and trigger renewed periapical inflammation. In this context, the solubility of CSS emerges not only as a laboratory concern but also as a clinically relevant factor that may influence long‐term prognosis. This consideration extends beyond technical debate and touches the fundamental purpose of root canal filling materials, prompting a re‐evaluation of clinical priorities between promoting biological activity and maintaining mechanical stability. Perhaps the key lesson offered by CSS is not to prioritise one property over another but to recognise the importance of balance, understanding that stability and bioactivity are complementary rather than opposing aspects.

## 4 Knowledge Gaps and Future Directions

This brings us to a broader issue that shapes all previous discussions: the current state of clinical research. In this regard, CSS offer both promise and uncertainty, since although the number of clinical investigations is slowly increasing, the evidence base remains limited and long‐term data are still scarce. Available clinical studies and meta analyses consistently show that CSS perform similarly to traditional sealers (Sabeti et al. 2024; Zamparini et al. 2024; Kangseng et al. 2025; Seog et al. 2025), and this equivalence holds across different pulp and periapical conditions (Sabeti et al. 2024; Kangseng et al. 2025; Seog et al. 2025). They have also been associated with lower short‐term postoperative pain during the first 24 h compared with resin‐based sealers, although pain levels converge within 48–72 h (Ensinas et al. 2025; Mahmoud Hamdy Abada et al. 2025). These findings must be interpreted alongside considerations related to the filling technique. A thicker sealer mass, as seen in single‐cone approaches, may increase exposure to dissolution and potentially amplify sealer‐related changes over time, while techniques that use more core material and less sealer may reduce these effects and promote more stable long‐term behaviour. Even so, clinical evidence shows that the short‐term and long‐term success of CSS used with the single‐cone technique remains comparable to outcomes achieved with resin‐based sealers placed using warm vertical compaction (Sabeti et al. 2024; Alzoubi et al. 2025; Kangseng et al. 2025; Seog et al. 2025). In fact, the strongest predictors of failure associated with CSS included the presence and size of preoperative periapical lesions and extensive sealer extrusion, although most extrusions did not lead to clinical symptoms or persistent radiolucency (Kim et al. 2022; Bamrungwong et al. 2025; Kangseng et al. 2025). Other variables such as patient age, gender, tooth type, number of visits, filling technique, and quality of the coronal restoration have not shown a significant influence on treatment outcomes (Kim et al. 2022; Bamrungwong et al. 2025; Bani‐Younes et al. 2025; Kangseng et al. 2025; Seog et al. 2025).

Although the current evidence shows that CSS perform as well as traditional sealers in clinical settings, a careful appraisal of the existing studies remains essential. The meta‐analyses of CSS studies (Sabeti et al. 2024; Zamparini et al. 2024) included randomised and prospective clinical trials, which increases the reliability of the findings. However, most available clinical studies included follow‐up periods shorter than 2 years, which restricts the ability to assess long‐term outcomes and late failures. Substantial heterogeneity in filling techniques, sealer formulations, and operator expertise further reduces the generalisability of pooled data, while the number of high‐quality randomised controlled trials remains small and several included studies present moderate risk of bias. Outcome reporting is also inconsistent, with varying definitions and measurement methods for success, survival, pain, and extrusion, thereby complicating comparison across studies. In summary, available meta‐analyses emphasised the need for well‐designed, prospective investigations with long‐term follow‐up to determine whether laboratory‐reported limitations translate into clinically meaningful effects. Taken together, these constraints indicate that although short‐term outcomes of CSS appear similar to those of conventional materials, the current evidence base is not yet sufficient to determine their long‐term clinical performance or to fully address concerns about their material properties.

This perspective encourages clinicians and researchers to look beyond short‐term results, question long‐held assumptions, and adopt a patient‐focused approach in which both scientific investigation and clinical decisions are guided by careful, long‐term observation, recognising that understanding the true effectiveness and lasting impact of these materials requires sustained evaluation rather than reliance on immediate outcomes alone. In the absence of robust, long‐term studies extending across multiple years, the behaviour of CSS over time remains only partially understood. We continue to operate in a space between promise and uncertainty, recognising both their potential to accelerate healing and the caution required regarding their long‐term stability.

These uncertainties are made even greater by the fact that CSS do not have a standard formulation. Although they fall under the same category, compositions of CSS can differ widely, including variations in radiopacifiers, additives, and the amounts of bioactive components (Radwanski et al. 2025), many of which are not fully disclosed to clinicians. These differences in components may be important because they can affect key properties of the material (Raman and Camilleri 2024), including its setting behaviour, ion release, solubility, and long‐term stability. In practice, this means that each formulation may behave differently in the clinic, and results observed with one sealer cannot automatically be applied to another. The lack of transparency makes it difficult for clinicians to interpret outcomes and for researchers to obtain reliable evidence, adding yet another layer of complexity to understanding these materials.

The present reflections remind us that scientific progress rarely follows a straight path and that every innovation brings both opportunities and responsibilities. To fully understand these materials, we must look past short‐term outcomes and investigate their long‐term effects, acknowledging that the true measure of healing is revealed over years, not months. This recognition reinforces the need for broader clinical research, including retrospective and prospective studies with long‐term follow‐up periods of at least 5 years that use CBCT‐based assessment to reflect how these materials perform in real clinical situations. It also shows the importance of clear information about material formulations, consistent reporting across studies, and experimental models that better reproduce the biological environment and functional behaviour of these sealers. Without such efforts, research risks producing data without meaningful insight, leaving fundamental questions unresolved. As endodontic filling materials continue to evolve, only through careful, methodologically sound, and clinically relevant research can we ensure that innovation translates into lasting benefits, aligning scientific advancement with improved patient outcomes.

## Author Contributions

**Emmanuel João Nogueira Leal da Silva:** conceptualization, writing – review and editing (lead). **Marco A. Versiani:** conceptualization, writing – review and editing (lead).

## Funding

The authors have nothing to report.

## Ethics Statement

The authors have nothing to report.

## Conflicts of Interest

The authors declare no conflicts of interest.

## Data Availability

The authors have nothing to report.
